# Osteomyelitis of the Mandibular Symphysis Caused by Brown Recluse Spider Bite

**Published:** 2008-08-28

**Authors:** Deepak K. Naidu, Rami Ghurani, R. Emerick Salas, Rudolph J. Mannari, Martin C. Robson, Wyatt G. Payne

**Affiliations:** Institute for Tissue Regeneration, Repair, and Rehabilitation, Bay Pines VA Medical Center, Bay Pines, Florida, and the Division of Plastic and Reconstructive Surgery, University of South Florida, Tampa, Florida; Presented in part at the Symposium for Advanced Wound Care, Tampa, Florida, April 29, 2007.

## Abstract

**Background:** Brown recluse spider bites cause significant trauma via their tissue toxic venom. Diagnosis of these injuries and envenomation is difficult and many times presumptive. Treatment is varied and dependent upon presentation and course of injury. **Materials and Methods:** We present a case of a previously unreported incidence of osteomyelitis of the mandible as a result of a brown recluse spider bite. A review of the literature and discussion of diagnosis and treatment of brown recluse spider bites are presented. **Results:** Osteomyelitis of the mandible causing a chronic wound was the presenting finding of a patient with a history of spider bite and exposure to brown recluse spiders. Operative debridement and wound closure resulted in successful treatment. Brown recluse spider envenomation varies in its presentation and treatment is based on the presenting clinical picture. **Conclusion:** Treatment regimens for brown recluse spider bite envenomation should include the basics of wound care. Systemic antibiotics, topical antimicrobials, dapsone, and surgical debridement are valuable adjuncts of treatment, as indicated, based on the clinical course.

Brown recluse spider bite envenomation can be a significant traumatic injury. Probably overreported, brown recluse spider bites and their resultant tissue injury patterns are well documented; however, treatment regimens are somewhat controversial. We present an unusual case of a presumed brown recluse spider bite injury of the face, which developed into chronic osteomyelitis of the mandible, and discuss the evaluation and further management of this interesting clinical presentation.

## CASE REPORT

**Figure 1:**
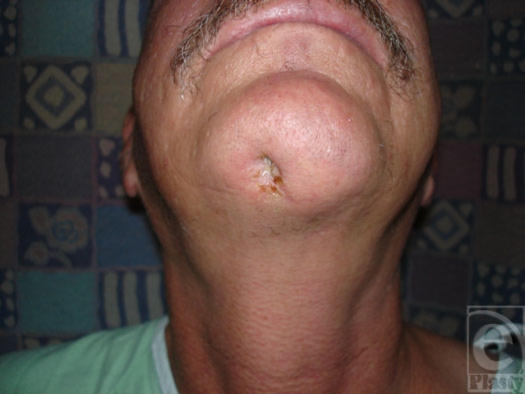
Preoperative photograph of presenting wound.

**Figure 2:**
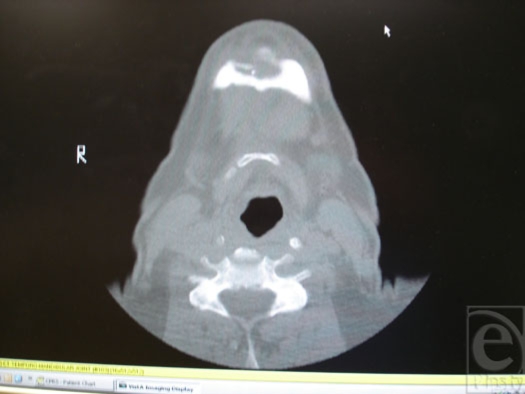
Computed tomographic scan demonstrating infection of mandibular symphyseal bone.

A 52-year-old white male presented with a 6-month history of a tender, open wound in the submental area. The chronic wound had been draining serous and purulent material for 4 to 6 weeks prior to evaluation. The wound began as a “pimple” on his chin that progressed in a few days to erythema, skin breakdown, and then, eventually, purulent drainage. The patient related a history of having encountered multiple “fiddleback” spiders at his place of work. He also claimed to have suffered multiple spider bites on the extremities in the past, all of which healed without sequelae. Physical examination was significant for a 5-mm open wound of the mandible, slightly to the right of the midline of his chin (Fig 1). The area was tender and a small amount of murky fluid was expressed. The wound probed to bone, using a sterile hemostatic clamp. There was no palpable regional lymphadenopathy. Computed tomography revealed a large bony cavity eroding through the inferior cortex of the mandibular symphysis anteriorly and abutting the lingual cortex. The mandibular integrity was intact with no evidence, clinically or radiographically, of a pathological fracture (Fig 2). The bony destruction appeared chronic, as evidenced by the sclerotic margins of the cavity. *Differential diagnosis* included possible odontogenic infection, neoplastic process, and traumatic wound infection. An odontogenic infection was excluded because of the viability of associated dentition and lack of demonstrable dental pathology. As a soft tissue or bony neoplasm appeared unlikely because of the smooth, sclerotic margins seen on computed tomographic scan and the lack of a demonstrable soft tissue lesion, this was attributed most likely to a wound infection secondary to traumatic injury.

**Figure 3:**
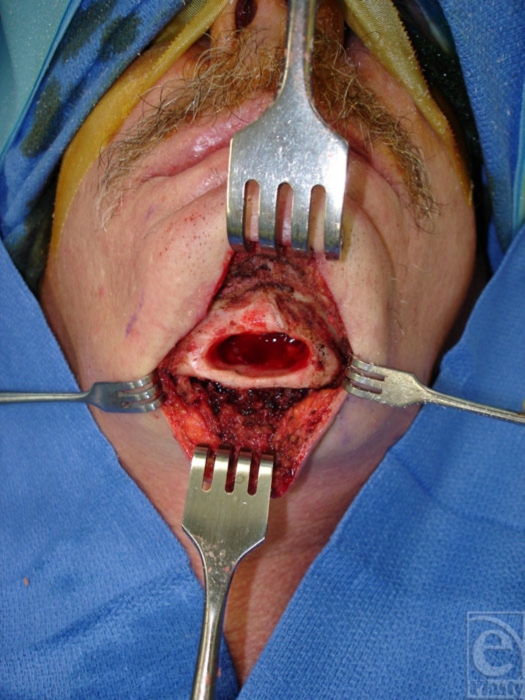
Intraoperative photograph of debrided, stable mandibular symphysis.

**Figure 4:**
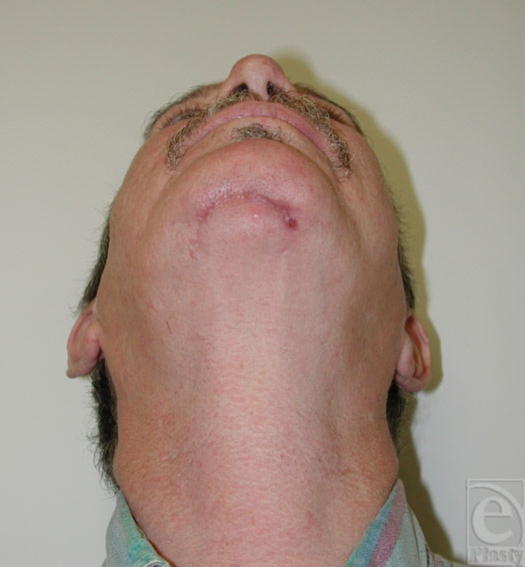
Postoperative photograph demonstrating healed, stable wound.

Operative debridement was planned with a presumptive diagnosis of traumatic infection. The patient underwent excision of the wound and the chronic granulation/soft tissue inflammatory reaction within the bony cavity. The bone cavity was aggressively curetted and debrided to healthy bleeding bone (Fig 3). The mandibular symphysis was found to be extremely stable with adequate stock of bone to maintain its integrity without the need for mechanical reinforcement, either by plating or bone graft. The soft tissue was extensively mobilized and reapproximated in layers. The pathology report revealed chronic osteomyelitis. At 6 weeks follow-up, the wound had healed without complication or further sequelae (Fig 4).

## DISCUSSION

The brown recluse spider, *Loxosceles recluse* is the most common of the *Loxosceles* species in the United States. Clinical sequelae of *Loxosceles* bites were described in the literature as early as 1879.^1^ According to the Centers for Disease Control and Prevention, approximately 10,000 spider bites are annually reported to poison control centers, of these, 1835 bites were attributed to the brown recluse in 1994.^2^ The brown recluse spider mainly populates the southern central states, due to a preferred habitat of mild climates.^3^ The spiders prefer dark, quiet environments such as closets, basements, attics, and sheds. Brown recluse spiders do not usually bite unless provoked or threatened. The spider is generally 1 to 5 cm in length and has a tan to light brown color. Distinguishing features include fiddle-shaped brown markings on its dorsum and characteristic 3 dyads (6 eyes as opposed to the usual 8 for most spiders).^4^ Brown recluse spiders commonly bite exposed lower or upper extremities, but bites to the face have been reported.^5^

Brown recluse spider bite injuries cause envenomation leading to major tissue destruction.^6^ Pain and erythema are apparent within the first few hours after the bite. Progression to skin necrosis with purplish blue cyanotic bullae within 24 to 48 hours soon follows. The site of injury undergoes a cycle of erythema, ischemia, and thrombosis, referred to as the “red, white, and blue sign.”^7(p565)^ This is due to brown recluse venom components, including hyaluronidase, elastase, sphingomyelinase D, lipase, and serum amyloid protein.^8^ Each of these enzymes contribute to the extent of tissue necrosis.^8^ The envenomation attracts polymorphonuclear cells, which further propagate the necrosis. The polymorphonuclear cells degranulate within the vasculature leading to vessel thrombosis followed by tissue ischemia. The underlying area of necrosis is generally extensive in comparison to the minimal surface lesion as seen in our patient. The clinical picture may progress to signs of fever, chills, malaise, vomiting, and arthralgias. There have even been reports of brown recluse spider bite envenomation leading to multisystem organ failure and death.^9^

The diagnosis and treatment of brown recluse bites is controversial: a classification system proposed by Anderson has been favored by expert entomologists to facilitate the diagnosis and, perhaps, to decrease overdiagnosis in nonendemic areas.^6^^,^^10^^,^^11^ A number of entomologists have collected data from reported brown recluse spider bites, but they noted that positive identification of the spider has been absent.^11^^,^^12^ Laboratory tests using enzyme-linked immunosorbent assay techniques have been developed in attempt to accurately diagnose brown recluse envenomation but none have been widely accepted or approved for clinical use.^13^

The differential diagnosis of brown recluse spider bite envenomation includes thromboembolic disease; focal vasculitis; drug reactions; pyoderma gangrenosum; Lyme disease, bacterial, viral, or fungal infections; neoplasms; chemical burns; and factitious injections.^4^^,^^5^ Attempts to exclude other potential causes before attributing necrotic skin lesions to the probably overused diagnosis of brown recluse bite should be made. Nonendemic regions are especially unlikely to encounter the problem and other more probable diagnoses should be entertained.

Treatment includes basic wound care measures, such as rest, ice compresses, and elevation of the involved extremity. Antibiotics are typically not indicated unless there is skin breakdown with secondary infection caused by typical skin flora. *Dapsone*, a leukocyte inhibitor, has shown some promise, if administered early. Dapsone's efficacy is based on its inhibitory effect on the polymorphonuclear cells that infiltrate and further propagate thrombosis, leading to further ischemia and tissue necrosis. Dapsone is usually reserved for severe cases because of its adverse effects, including dose-related hemolysis, agranulocytosis, aplastic anemia, cholestatic jaundice, and methemoglobinemia.^14^

Operative intervention is indicated when frank abscess formation occurs, tissue necrosis is extensive or severe, or deep vital structures are involved or exposed.^5^^,^^7^ Incision and drainage of a defined abscess is always indicated; however, as intense tissue inflammation and edema are more common findings than discrete abscesses, indiscriminate incision without the presence of an underlying abscess should be avoided. Debridement of necrotic tissue is necessary to avoid the negative metabolic effects of the breakdown products of cell death on wound healing. Deep exposed structures such as bone or vital tissues may require soft tissue coverage by an appropriate flap or graft after adequate wound bed preparation.

Our patient presented with a prolonged course of a chronic, open wound with deep soft tissue and bone involvement. There was chronic infection manifested by osteomyelitis of the mandible. Appropriate soft tissue debridement and bone debridement were performed and primary wound closure was obtained. Long-term follow-up demonstrated resolution of the chronic draining wound with surgical management and antibiotic therapy. Although the spider is not considered endemic to Florida, the patient did identify environmental exposure and clinical presentation consistent with a brown recluse spider bite.

## CONCLUSION

Brown recluse spider bites and envenomations may be overreported. The course of injury may vary from mild erythema to frank necrosis of soft tissue and bone. The treatment regimen should include the basics of wound care and consideration of systemic antibiotics and topical antimicrobials as indicated. Dapsone may be of value if given early in the course of treatment. Surgical intervention is warranted in cases of abscess formation, extensive tissue necrosis, or deep tissue involvement. Injuries to the face are rare but follow the course of injury to other more common areas of the body.

## Acknowledgment

The authors thank Chris R. Payne, Major, USAF (retired), for assistance in the preparation of photographs for this article. There was neither financial support nor do any authors have proprietary interest in any product, device, or method discussed in this article.
